# Exploring the relationship of clinical walking tests with 8-months inertial measurement unit (IMU)-based real world mobility tracking in stroke and spinal cord injury survivors

**DOI:** 10.1186/s42466-025-00386-z

**Published:** 2025-05-09

**Authors:** Andreas Hug, Tamara Spingler, Viola Pleines, Laura Heutehaus, Mircea Ariel Schoenfeld, Björn Hauptmann, Jürgen Moosburger, Roland Thietje, Oliver Pade, Wolfgang Rössy, Klaus Stecker, Jochen Klucken, Tiziana Daniel, Michel Wensing, Cornelia Hensel, Rüdiger Rupp, Norbert Weidner

**Affiliations:** 1 Spinal Cord Injury Center, Heidelberg University Hospital, Schlierbacher Landstr. 200a, 69118 Heidelberg, Germany; 2 Kliniken Schmieder, Speyererhofweg 1, 69117 Heidelberg, Germany; 3 Department of Behavioral Neurology, Leibniz Institute for Neurobiology, 39118 Magdeburg, Germany; 4 Department of Neurology, Otto von Guericke University Magdeburg, 39120 Magdeburg, Germany; 5 Neurological Center, Segeberger Kliniken, Hamdorfer Weg 3, 23795 Bad Segeberg, Germany; 6 Department Performance, Neuroscience, Therapy and Health, Medical School Hamburg, Am Kaiserkai 1, 20457 Hamburg, Germany; 7 Medical Rehabilitation Center for Spinal Cord Injured “Heinrich-Sommer-Klinik”, Paulinenstr. 132, 75323 Bad Wildbad, Germany; 8 Spinal Cord Injury Center, BG Klinikum Hamburg, Bergedorfer Straße 10, 21033 Hamburg, Germany; 9 Klinikum Bad Bramstedt GmbH, Klinik für Neurologische Rehabilitation, Oskar-Alexander-Str. 26, 24576 Bad Bramstedt, Germany; 10 St. Rochus-Kliniken, Klinik für Neurologie, Sankt-Rochus-Allee 1, 76669 Bad Schönborn, Germany; 11 August-Bier-Klinik, Diekseepromendade 7-11, 23714 Bad Malente, Germany; 12 Luxembourg Institute of Health (LIH), Strassen, Luxembourg; 13 Department of General Practice and Health Services Research, Heidelberg University Hospital, 69120 Heidelberg, Germany

**Keywords:** Spinal cord injury, Stroke, Everyday activity, Mobility, Walking distance, Wheelchair distance, Clinical walking tests, Ecological validation

## Abstract

**Background:**

Mobility is crucial for participation and quality of life in individuals with sensorimotor impairments, yet scientific evidence on its course in real-world settings is limited. So-called wearables for measuring physical activity might help to overcome this knowledge gap allowing daily measurements of mobility. The aim of the present study is to examine the relationship between clinical walking tests and inertial measurement unit-based mobility tracking in the community setting of stroke and spinal cord injury (SCI) survivors.

**Methods:**

At a single observational time point, the precision of the activity tracker was evaluated in a standardized parcours in healthy subjects and stroke or SCI survivors (*n*=57). This was followed by a multicenter observational cohort study (*n*=116 participants), in which the mobility of stroke and SCI survivors was assessed over 8 months immediately after discharge from acute inpatient rehabilitation. Daily distances covered in the community setting were recorded using the activity tracker. Established walking tests—including the 10-meter walk test (10MWT) and the timed up and go test (TUG)—were conducted at baseline, as well as at 4- and 8-month follow up visits. The relationship between daily distances in the ambulatory setting and 10MWT or TUG performance at discrete study visits (baseline, 4 months (midterm), and 8 months (final) after hospital discharge) was analyzed using regression models.

**Results:**

The precision of the activity tracker in measuring covered distance in a standardized parcours varied by mobility type. The highest precision was achieved in manual wheelchair users (deviation from zero: -1.5±1.03% (*p*=0.15) while the least favorable precision was observed in participants with SCI and significant walking impairment (-14.6±2% (*p*<0.001). The widely used 10MWT speed showed a relationship with the ambulatory daily distance. The regression coefficients [m/(1m/s)] were: 874 (95% CI: 578-1171) at baseline (*p*<0.001), 895 (95% CI: 614-1176) at midterm (*p*<0.001), and 824 (95% CI: 537-1112) at the final visit (*p*<0.001). Interestingly, in the category of good walkers with the most favorable walking speeds the daily covered distance unmasked distinct subgroups with shorter and longer daily distances.

**Conclusions:**

For SCI and stroke survivors, especially medium to fast walkers, activity tracking in real-world settings adds valuable insight beyond clinical walking tests. Clinical studies on rehabilitative interventions for mobility improvement should consider real-life daily distance as a key endpoint.

**Supplementary material:**

The online version of this article (10.1186/s42466-025-00386-z) contains supplementary material, which is available to authorized users.

## Background

Regaining mobility is a primary goal in neurological rehabilitation, as it significantly impacts quality of life and social participation (Ezekiel et al., 2019). However, little is known on how individuals with sensorimotor impairments—whether walking or using a wheelchair—actually move in their daily lives after discharge from inpatient rehabilitation. This is particularly important because evidence suggests that mobility levels often decline once patients transition to community-based care, as seen in stroke survivors (Meyer et al., 2015).

Long-term studies assessing real-world mobility, measured in distance covered over months, are rare. Such data could provide valuable insights into both activity levels and social participation (Corbett et al., 2018). Most existing research relies on short-term activity monitoring (4-7 days), primarily counting steps or general activity units (Duncan et al., 2011; Hale et al., 2008; Kluding et al., 2013; Lemay et al., 2012; Mahendran et al., 2016). With recent advancements in wearable technology for continuous activity tracking, there is now an opportunity to bridge this knowledge gap, especially as these devices become more widely used (Thompson, 2023).

Traditionally, mobility outcomes are assessed using standardized walking tests, such as the 10-meter walk test (10MWT), at specific time points following conditions like stroke or spinal cord injury (SCI). While these tests effectively measure mobility capacity under controlled conditions and correlate with short-term step counts (Bowden et al., 2008), their ability to reflect long-term, real-world mobility after discharge remains unclear. This is particularly relevant for patients with moderate to good walking ability after stroke, where the ecological validity of standardized clinical walking tests remains questionable (Stellmann et al., 2015). For SCI survivors, no such data exist.

This study aimed to explore the relationship between standardized clinical walking tests and decentralized daily tracking of ambulation in the community-environment after inpatient rehabilitation in stroke and SCI survivors (Hug et al., 2021). We compared real-world mobility data (“what the patient does”) with established gait tests (“what the patient can do”), such as the 10MWT and Timed Up and Go (TUG) test. A wearable IMU device, previously validated for mobility tracking in elderly individuals and patients with Parkinson’s disease or multiple sclerosis (Barth et al., 2015; Flachenecker et al., 2020; Klucken et al., 2011) - was tested in stroke and SCI survivors, as well as healthy controls. The device was then used to record daily walking distances for eight months post-discharge. We analyzed these data for relationships with standardized walking tests (10MWT, TUG) performed in the clinical settings at three time points (0, 4, and 8 months after discharge).

## Methods

The study was conducted as part of the NeuroMoves project (Hug et al., 2021) and approved by the Ethics Committee of the Medical Faculty of Heidelberg University (Approval-IDs: S-084/2020, S-858/2019).

### Study design and setting

In a first step, we used a cross-sectional design to assess the sensor precision for a standardized distance. The sensor was previously validated for mobility tracking in elderly individuals and patients with Parkinson’s disease or multiple sclerosis (Barth et al., 2015; Flachenecker et al., 2020; Klucken et al., 2011). Participants with incomplete SCI or stroke, treated at the Spinal Cord Injury Center and Kliniken Schmieder in Heidelberg, Germany, were enrolled, along with a control group of non-disabled subjects (NDS). Participants completed a fixed-length indoor course (parcours) of 250 (SCI, NDS) or 188 meters (stroke, NDS), differing due to institutional conditions. The parcours included three sections: straight line, semicircle, figure-of-eight. In a second step, a multicenter (8 study sites) observational cohort design was used to follow up study participants with stroke or SCI in the community setting for a fixed period of 8 months immediately after discharge from acute inpatient rehabilitation.

### Study participants

For the first step, subjects were included if they were attributable to one of the following cohorts: Manual wheelchair users (stroke or SCI), electric wheelchair users (stroke or SCI), pedestrians with SCI, pedestrians with stroke, or NDS. A target sample size of *n*=10 per group was chosen based on theoretical considerations to best represent the mobility types in the NeuroMoves project, without formal sample size calculations.

For the second step, patients (18-85 years of age) diagnosed with SCI or stroke according to the International Classification of Diseases (ICD)-10 criteria were eligible. Participants were screened, enrolled, and instructed regarding device use by study personnel at the clinical sites towards the end of the acute inpatient rehabilitation period. Sensor-based measurements started immediately after discharge.

Any study-related procedure was performed only after obtaining written informed consent from participants.

### Data sources/ measurements

This study used the Mobile GaitLab system (Portabiles HealthCare Technologies, Nuremberg, Germany), comprising a sensor device for raw data acquisition and a tablet computer for data processing and backup. The IMU-based sensor includes a 3-axis gyroscope and a 3-axis accelerometer, recording data at 100 Hz. The device was mounted differently depending on mobility type: on the right wheel near the axle for wheelchair use (using adhesive tape) and on the dorsum of the foot for walking (using a clip holder attached to shoelaces or Velcro). Suitable for home monitoring (Barth et al., 2015) the sensor stored raw data locally during measurements and transferred it to the tablet during charging. Computational algorithms processed the data offline to identify mobility type and calculate distances traveled using proprietary software.

Demographic, clinical, and walking tests data of the NeuroMoves cohort were collected at three discrete clinical visits at each participating clinical site (baseline, 4 months (midterm), 8 months (final)). Both, the 10MWT (Rossier & Wade, 2001) and the TUG (Podsiadlo & Richardson, 1991) were performed at each clinical visit. Only participants with walking capability were included in this part of the study. At each visit, the Functional Independence Measure (FIM) was performed as a measure of functional independence. The total FIM score ranges from 18 to 126, with the motor FIM subscore ranging from 13 to 91 and the cognitive FIM subscore ranging from 5 to 35 (Granger et al., 1993). Daily walking distances were measured using the activity tracker. The tracker was recharged and its data backed up each evening. Raw data were transferred to a home tablet, preprocessed, and uploaded daily to a cloud-based study management system (SMS). Data uploads were monitored via software algorithms and regular phone contact with study personnel.

### Variables

In participants with stroke, the degree of disability was assessed by the National Institute of Stroke Scale (NIHSS) (Brott et al., 1989) and the modified Rankin Scale (mRS) (Rankin, 1957). Participants with SCI were assessed neurologically in accordance with the International Standards for Neurological Classification of Spinal Cord Injury (ISNCSCI) (Kirshblum et al., 2011; Rupp et al., 2021). This examination determined the American Spinal Injury Association (ASIA) Impairment Scale (AIS) grade and the neurological level of injury (NLI). The NLI classified participants as having paraplegia (T1 and below) or tetraplegia (C0–C8). Age and sex were included as demographic variables. The self-selected speed for the parcours was calculated as: parcours length ÷ elapsed time [m/s]. Prior to completing the parcours as a pedestrian, the TUG (Podsiadlo & Richardson, 1991) was performed to estimate the propensity to fall. As measure of precision of the sensor-based calculated distance, the relative deviation from the actual parcours length was calculated according to the formula: $\frac{\text{Sensor-based distance}-\text{Actual parcours length}}{\text{Actual parcours length}}\times 100\%$.

The result of the 10MWT was reported as velocity in m/s. For the TUG test, the time required in seconds was analyzed. For the daily walking distance, the tracked discrete walking activities were summed up. For univariate analyses, a grand mean of daily distance per subject was calculated by averaging the sum of all recorded daily distances over the total number of follow-up days.

### Statistical methods

Where applicable, variables were aggregated by reporting absolute and relative frequencies for nominal variables. Continuous variables were summarized by calculation of mean values. The standard deviation was chosen as a measure of dispersion. The ordinal-scaled numerical NIHSS variable was also summarized by calculating the mean, assuming pseudo-continuous scaling. The ordinally scaled numerical mRS variable was summarized by absolute and relative frequencies. A mixed linear model was used to examine relationships between relative deviation from the actual parcours length, study population (SCI, stroke, or NDS), mobility type, and parcours length. Marginal means were estimated to compare mean relative deviations across mobility type and study population groups. In the NeuroMoves cohort, our primary aim was to evaluate the direction and magnitude of the relationship between standardized clinical walking tests (10MWT, TUG) and real-world mobility measures derived from inertial measurement units (IMUs) using linear regression models. Ordinary least squares regression was performed using the base R function *lm()*, and linear mixed-effects models were fitted using the *lme4* package (Bates et al., 2015). The exact parameterization of these statistical models is provided in the supplementary appendix. All regression analyses were assessed for outliers using studentized residuals. Observations with studentized residuals exceeding an absolute value of 3 were considered outliers and excluded from the final regression analysis.This threshold was chosen based on common statistical practices for detecting influential observations in regression analyses. Data wrangling and statistical analyses were performed with tidyverse packages (Wickham et al., 2019) using the IDE RStudio with R version 4.4.0–“Puppy Cup”.

## Results

### Precision of sensor-based distance calculation

For the precision analysis on a standardized parcours, 57 participants (20 stroke, 24 SCI, 13 NDS) were recruited between August and December 2020. Observations were categorized by mobility type—electric wheelchair (EW), mechanical wheelchair (MW), or walking—based on how participants completed the parcours. Since some individuals used multiple mobility types, a total of 81 mobility-type observations were recorded (32 for the 188.3m parcours and 49 for the 250.4m parcours). The subject characteristics based on sample population by mobility type combinations are shown in Table 1. Walking speed in the parcours was significantly faster in NDS as compared to individuals with stroke (pairwise difference [mean ± standard error of the mean (SEM)] 0.3±0.07 m/s, *p*=0.002) and SCI (pairwise difference 0.8±0.11 m/s, *p*<0.001), respectively. In participants with SCI, the walking speed was slower as compared to participants with stroke (pairwise difference -0.5±0.11 m/s, *p*<0.001). TUG times were shorter in NDS as compared to stroke survivors (pairwise difference -6±1.1 s, *p*<0.001) and SCI participants (pairwise difference -10.7±1.73 s, *p*<0.001), respectively. Participants with SCI had longer TUG times versus those with stroke (pairwise difference 4.7±1.77 s, *p*=0.0304).

**Table 1:** Overview of characteristics of study participants for the sensor validation step

	NDS		SCI			Stroke
Characteristic	MW N = 11	WALK N = 24	EW N = 7	MW N = 14	WALK N = 5	WALK N = 20
*Sex, n (%)*						
female	6 (55)	14 (58)	0 (0)	3 (21)	0 (0)	10 (50)
male	5 (45)	10 (42)	7 (100)	11 (79)	5 (100)	10 (50)
Age [years], Mean (SD)	25 (4)	25 (4)	62 (9)	48 (18)	63 (13)	55 (14)
Parcours speed [m/s], Mean (SD)	1.2 (0.3)	1.4 (0.1)	0.9 (0.2)	0.6 (0.1)	0.6 (0.2)	1.1 (0.3)
TUG [sec], Mean (SD)		5.9 (1.1)			16.6 (3.4)	13.9 (10.1)
*AIS, n (%)*						
	11 (100)	24 (100)				20 (100)
A			4 (57)	4 (29)	0 (0)	
B			0 (0)	2 (14)	0 (0)	
C			2 (29)	2 (14)	0 (0)	
D			1 (14)	6 (43)	5 (100)	
*NLI, n (%)*						
	11 (100)	24 (100)				20 (100)
paraplegia			2 (29)	11 (79)	3 (60)	
tetraplegia			5 (71)	3 (21)	2 (40)	
NIHSS, Mean (SD)						2 (3)
*mRS, n (%)*						
1						6 (30)
2						8 (40)
3						5 (25)
4						1 (5.0)

NDS, non-disabled subjects; SCI, spinal cord injury; EW, electrical wheelchair; MW, manual wheelchair; WALK, walking;TUG, Timed-up-and-Go Test; AIS, American Spinal Injury Association Impairment scale; NLI, neurological level of injury; NIHSS, National Institute of Health Stroke Scale; mRS, modified Rankin score; SD, standard deviation.

**Fig. 1:**
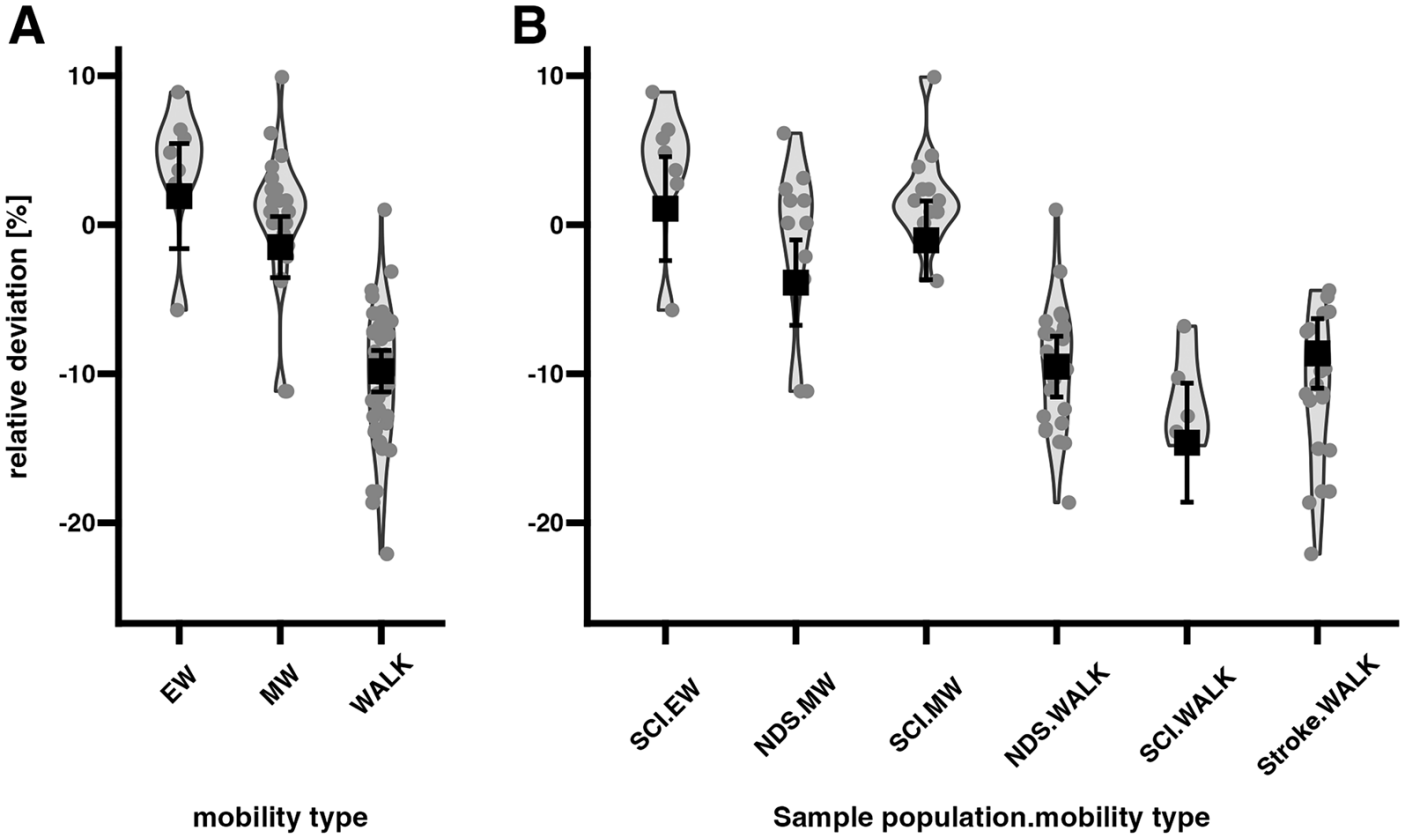
Observed relative deviation of sensor-based calculated distance from actual parcours length for each study participant (gray dots) grouped by (**A**) mobility type and (**B**) sample population by mobility type interaction. Black squares with error bars represent model-based means ± 2× SEM. Abbreviations: EW=electric wheelchair, MW=manual wheelchair, SEM=Standard error of mean

In descriptive univariate analyses, the mobility type MW was associated with the smallest relative deviation from the actual length of the parcours (Fig. 1A). In multivariate analyses, the actual parcours length effect was statistically significant and positive (0.06 %/m (95% CI 0.02, 0.10; *p*<0.002)). Hence, in the context of negative relative deviations in walking participants, the degree of underestimation diminishes as walking distances increase. Accounting for this actual parcours length effect revealed the following relative deviation estimates in relation to mobility type (hypothesis test: difference from zero): EW: 1.9±1.76% (*p*=0.28). MW: -1.5±1.03% (*p*=0.15). Walking: -9.8±0.7% (*p*<0.001) (Fig. 1A).

The sensor-based calculated walking distance was uniformly lower than the parcours length across all study populations (NDS: -9.5±1.02% (*p*<0.001); SCI: -14.6±2% (*p*<0.001); Stroke: -8.6±1.17% (*p*<0.001) (Fig. 1B, Table 2). Sensor-based calculated distances in walkers with SCI demonstrated more negative relative deviation (higher degree of underestimation) of the actual parcours length as compared to NDS (-5.1±2.24% (*p*=0.026) and stroke survivors (-6±2.5% (*p*=0.02)), respectively (Fig. 1B). Sensor-based calculated distance estimates between NDS and people with stroke were not statistically different.

**Table 2:** Relative deviation from actual parcours length

Sample population	Mobility type	Relative deviation [%]	SEM	p-value
NDS	EW			
SCI	EW	1.1	1.74	0.537
Stroke	EW			
NDS	MW	–3.9	1.43	0.008
SCI	MW	–1.0	1.32	0.431
Stroke	MW			
NDS	WALK	–9.5	1.02	<0.001
SCI	WALK	–14.6	2.00	<0.001
Stroke	WALK	–8.6	1.17	<0.001

NDS, non-disabled subjects; SCI, spinal cord injury; EW, electrical wheelchair; MW, manual wheelchair; WALK, walking.

### Sensor-based real-world ambulatory daily distance

From May 2021 through July 2022 a total of *n*=116 participants with the ability to walk were recruited, had a baseline assessment, and were followed up at two scheduled time points (midterm at 4 months and final visit at 8 months). The clinical characteristics at baseline are shown in Table 3. The midterm visit took place on average (SD) 131 (15) days and the final visit 253 (21) days after the baseline visit (Table 4).

**Table 3:** Overview of characteristics of NeuroMoves study cohort

Characteristics	SCI, N = 39	Stroke, N = 77	*p*-value^1^
Sex, n (%)			0.35
female	15 (38)	23 (30)	
male	24 (62)	54 (70)	
Age [years], Mean (SD)	54 (14)	59 (12)	0.076
NLI, n (%)		NA	
Paraplegic	25 (64)		
Tetraplegic	14 (36)		
10MWT speed [m/s], Mean (SD)	0.6 (0.4)	0.8 (0.4)	0.028
TUG [sec], Mean (SD)	34.8 (31.0)	23.3 (20.7)	0.010
*AIS, n (%)*			
A	2 (5.1)		
C	9 (23)		
D	28 (72)		
NIHSS, Mean (SD)		3 (2)	
mRS, n (%)			
0		5 (6.6)	
1		9 (12)	
2		20 (26)	
3		38 (50)	
4		4 (5.3)	

^1^Pearson’s Chi-squared test; Wilcoxon rank sum test.

SCI, spinal cord injury; 10MWT, 10-meter walk test; TUG, Timed-up-and-Go Test; AIS, American Spinal Injury Association Impairment scale; NLI, neurological level of injury; NIHSS, National Institute of Health Stroke Scale; mRS, modified Rankin score; SD, standard deviation.

**Table 4:** Overview of available study assessments for the three NeuroMoves study visits

Characteristics	baseline, N = 116	midterm, N = 116	final, N = 116
Study visit day, Mean (SD)	0 (0)	131 (15)	253 (21)
10MWT speed [m/s], Mean (SD)	0.74 (0.41)	0.85 (0.45)	0.94 (0.49)
TUG [sec], Mean (SD)	27 (25)	22 (24)	22 (23)

10MWT, 10-meter walk test; TUG, Timed-up-and-Go Test; SD, standard deviation.

### Clinical walking tests

**Fig. 2:**
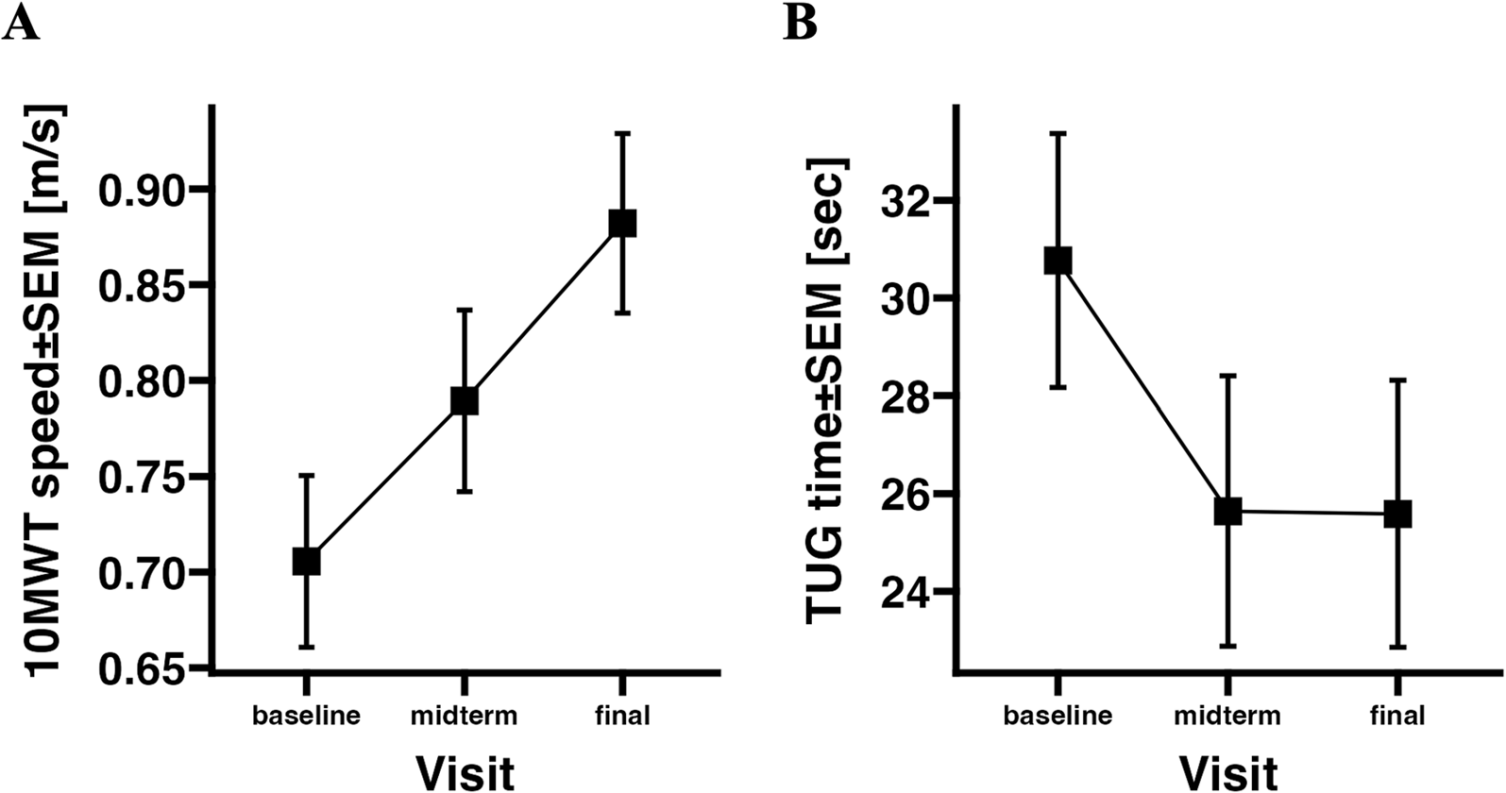
Walking tests results of the NeuroMoves cohort per visit time point (baseline=discharge from inpatient rehabilitation, midterm=4 month after baseline, final=8 month after baseline). **A** Mean ± SD of gait speed assessed with the 10 Meter Walk Test (10MWT). **B**: Mean ± SD of time to conduct the Timed up and Go Test (TUG)

Participants with stroke demonstrated, on average, faster walking speeds in the 10MWT and shorter TUG times compared to those with SCI (see Table 3). Overall, walking speed measured by the 10MWT improved over the observation period, increasing from a mean (SD) of 0.74 (0.41) m/s at baseline to 0.85 (0.45) m/s at midterm and 0.94 (0.49) m/s at the final visit (Table 4, Fig. 2A). The TUG times improved from am mean (SD) of 27 (25) s at baseline to 22 (24) s at midterm and remained constant thereafter with 22 (23) s at the final visit (Table 4, Fig. 2B). The differences in 10MWT speed were significant between all three time points; baseline to final visit: +0.17±0.03 m/s [mean difference ± SEM], *p*<0.001; baseline to midterm: +0.09±0.03 m/s, *p*=0.0017; midterm to final: +0.08±0.03 m/s, *p*=0.007. TUG times differed from baseline to midterm (-4.7±1.34 s, *p*=0.006) and baseline to final visit (-5.5±1.29 s, *p*<0.001), respectively. The TUG difference between midterm and final visit was not significant.

### Relationship between clinical walking tests and sensor-based data

**Fig. 3:**
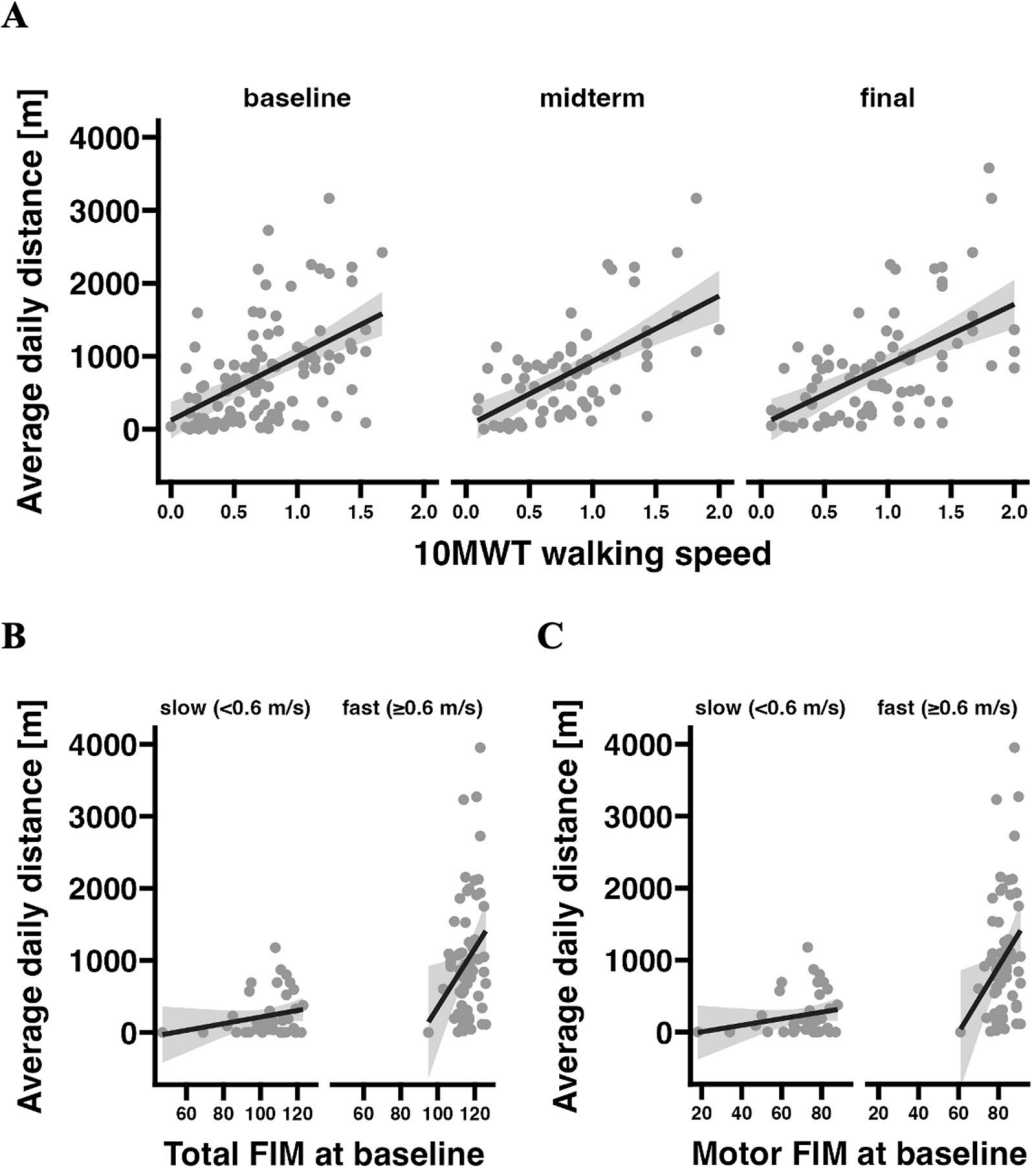
**A** Scatter plots with regression lines showing the relationship between average daily walking distance and 10 Meter Walk Test (10MWT) speed at baseline, midterm and final visits. **B** Scatter plots with regression lines illustrating the relationship between the baseline Functional Independence Measure (FIM) total score and average daily walking distance, stratified by walking speed category (slow: <0.6 m/s, fast: ≥0.6 m/s). **C** Scatter plots with regression lines illustrating the relationship between the baseline Functional Independence Measure (FIM) motor subscore and average daily walking distance, stratified by walking speed category (slow: <0.6 m/s, fast: ≥0.6 m/s)

Each of the three (baseline, midterm, final) visit-based discrete 10MWT measurements was associated with the ambulatory daily distance as analyzed by linear regression (Fig. 3A). The regression coefficients [m/(1m/s)] were: 874 (95% CI: 578-1171) at baseline (*p*<0.001), 895 (95% CI: 614-1176) at midterm (*p*<0.001), and 824 (95% CI: 537-1112) at the final visit (*p*<0.001). In linear mixed model regression analysis (accounting for the repeated measures design of daily distance), the 10MWT at baseline was a main explanatory variable of daily distance. As per 0.1 m/s 10MWT speed, the estimated daily distance was 83.9 m higher (95% CI: 49.7-118.2; *p*<0.001). Although baseline walking speed seems to be a good predictor of the daily distance covered, the residual variance still presents as quite large, particularly in subjects with faster walking speeds (see variance of data points in Fig. 3A). The additional analysis of the FIM score explained some residual variance in this context. We could observe an interaction effect between 10MWT speed and FIM in linear mixed model analysis (*p*=0.019). When dividing participants based on their walking speed into *slow* (<0.6 m/s) and *fast* (≥0.6 m/s) walkers (midrange speed of limited community walkers defined the cutoff (Bowden et al., 2008)), a significant positive correlation between the FIM total score and the daily distance covered could only be observed in *fast* walkers (Fig. 3B). Here, the motor FIM domain - as opposed to the cognitive FIM domain–contributed primarily to this association (Fig. 3C).

## Discussion

Our exploratory study provides insights into the clinical applicability of a wearable activity tracker in assessing real-world mobility in patients with stroke and SCI. The tested device demonstrated overall acceptable precision under controlled conditions, though variations were observed depending on the type of mobility (higher for wheelchair use than walking), the length of mobility sequences (greater for longer distances), and the study population (more precise in non-disabled than disabled walkers). The greatest measurement deviation was observed in individuals with SCI who walked, with an underestimation of approximately 15%. As the study design incorporated a commercially available activity tracker, the aim was not to improve its precision but rather to assess the extent of measurement error. Despite these variations, a strong relationship emerged between standardized walking tests (10MWT, TUG) and daily distance tracked over 8 months post-rehabilitation. However, among patients with moderate to good walking ability, significant variability remained unexplained, suggesting that clinic-based tests do not fully capture real-world mobility patterns.

Wearable devices currently available on the market face significant challenges in tracking mobility for individuals with neurological impairments (Kristoffersson & Lindén, 2022; Schneider et al., 2018). One of the primary limitations is their inability to autonomously distinguish between walking and wheelchair use, often requiring active user input. This restricts their ability to provide continuous mobility monitoring, particularly in patients transitioning between walking and wheeling. In our study we primarily aimed to use the tested IMU sensor (PHCT, Nürnberg) to capture both walking and wheelchair distances when attached to footwear. However, during our early assessment under controlled conditions, the footwear-attached sensor was unable to reliably switch between walking and wheeling, resulting in unreliable wheelchair distance measurements. Consequently, manual repositioning of the sensor to the wheelchair wheel using an additional plastic clip was required for reliable wheeling distance tracking.

The precision of activity trackers, such as the Fitbit®, is known to vary based on mobility type and sensor placement, with step counting being most precise during treadmill walking and least precise during low-intensity activities or when using walking aids (Alinia et al., 2017; Holubová et al., 2022). Hip-mounted sensors seem to be more precise than wrist-mounted trackers for step counts (Gaz et al., 2018). Since step recognition depends on sensor placement, we positioned the sensor on the dorsum of the foot to optimize walking distance measurement. Moreover, walking speed plays a critical role in sensor precision. Slower walking speeds have been associated with increased step-counting errors (Tedesco et al., 2019), and this was reflected in our findings: the SCI group, which walked at an average speed of 0.6 m/s—close to the lower threshold for community ambulation—had the highest sensor error. Stroke survivors, with a mean speed of 1.1 m/s (above the 0.8 m/s threshold for community ambulation (Bowden et al., 2008)), did not show significant measurement errors compared to the non-disabled sample. Although normative data in NDS show that walking speed decreases with increasing age (Bohannon & Andrews, 2011), this effect was not observed in our patient groups. This may be due to stroke or SCI—rather than age—being the primary cause of slower walking speeds, with participants with SCI and stroke walking slower than all age categories up to 79 years in the normative NDS dataset (Bohannon & Andrews, 2011). Additionally, the age distribution in our stroke and SCI populations was relatively narrow (54±14 years for SCI, 59±12 years for stroke), meaning that not all age categories were represented. As a result, the findings cannot be generalized to the full age range of individuals with mobility impairments following stroke or SCI.

An important clinical finding was that shorter walking distances were associated with greater sensor underestimation. This likely stems from step-detection algorithms that require a certain number of steps before initiating distance measurement. This underestimation is particularly relevant for individuals with slow walking speeds and those who frequently move indoors. Our study tested only two specific distances (188m and 250m). Hence, error extrapolation to shorter or longer distances remains speculative.

From a clinical perspective, our findings raise questions about the ecological validity of standardized walking tests such as the 10MWT. While the 10MWT has been validated against broader mobility assessments like the modified Rankin Scale (mRS) and the Stroke Rehabilitation Assessment of Movement (STREAM) (Cheng et al., 2021), its ability to reflect actual step counts or actual walking distances after hospital discharge has not been extensively studied (Fulk et al., 2017). This gap is important, as relying solely on in-hospital tests like the 10MWT may overlook significant clinical outcomes in everyday environments (Lord & Rochester, 2005). Ecological validity refers to the extent to which test results translate to real-world conditions (Suchy et al., 2024). In this context, our study demonstrates that higher 10MWT speeds in a clinical setting (“can do”) are associated with greater daily walking distances ("does do") in stroke and SCI survivors. This finding supports the use of the 10MWT results as a proxy for real-life walking distances in this population. However, this association was not uniform across all patients. Faster walkers exhibited greater variability in their real-world mobility, a finding consistent with studies in multiple sclerosis (MS), where individuals with higher 10MWT speeds did not necessarily translate this into greater daily walking distances (Stellmann et al., 2015). This discrepancy could be explained by residual functional limitations in activities of daily living: patients with good walking recovery but persistent deficits in other functional domains may remain more homebound despite their ability to walk faster. The high correlation of the 10MWT and the FIM score in the NeuroMoves population, particularly the motor FIM subscore, suggests a relationship with the functional recovery beyond locomotion. Given that the 10MWT only partially explains real-world mobility, continuous activity monitoring using wearable sensors could provide complementary data to enhance clinical assessments and rehabilitation planning.

Our study has several limitations. The reliance on a commercially available activity tracker, which did not allow for sensor precision enhancements, resulted in varying accuracy across mobility modes, particularly among SCI patients with walking impairments. Although we employed linear regression with adjustments for outliers, wide confidence intervals and signs of heteroscedasticity indicate that the precision of our estimates may be limited. Additionally, the relatively small sample sizes further constrain the generalizability of our findings.

## Conclusions

For individuals with stroke or SCI, particularly those with moderate to fast walking speeds, real-world activity tracking offers a valuable, clinically meaningful measure of mobility. Future rehabilitation studies could employ long-term mobility assessments—such as daily distance covered—as key outcome measures to evaluate the effectiveness of mobility-enhancing interventions.

## Data Availability

The datasets used and/or analysed during the current study are available from the corresponding author on reasonable request.

## Appendix

Below is the link to the electronic supplementary material. Supplementary file 1 (DOCX 591 kb)

## Abbreviations

10MWT: 10-Meter Walk Test
AIS: ASIA Impairment Scale
ASIA: American Spinal Injury Association
BG: Berufsgenossenschaft
CI: Confidence Interval
EW: Electrical Wheelchair
FIM: Functional Independence Measure
ICD: International Classification of Diseases
IDE: Integrated Development Environment
IMU: Intertial Measurement Unit
ISNCSCI: International Standards for the Classification of Spinal Cord Injury
m: meter
m/s: meter/second
mRS: modified Rankin Scale
MS: Multiple Sclerosis
MW: Mechanical Wheelchair
NDS: Non Disabled Subjects
NIHSS: National Institutes of Health Stroke Scale
SCI: Spinal Cord Injury
SD: Standard Deviation
SEM: Standard Error of the Mean
SMS: Study Management System
STREAM: Stroke Rehabilitation Assessment of Movement
TUG: Timed Up and Go Test
