# Supplementary material for: Exploring the relationship of clinical walking tests with 8-months inertial measurement unit (IMU)-based real world mobility tracking in stroke and spinal cord injury survivors
Source: Neurol Res Pract. 2025 May 9;7(1):30. doi: 10.1186/s42466-025-00386-z (PMC12063441; doi:10.1186/s42466-025-00386-z)
Supplement: Supplementary file 1 — (DOCX 591 kb) [file 42466_2025_386_MOESM1_ESM.docx]

# Supplementary statistical methods and results

The results of the applied statistical models were reported according to the R report package (Makowski et al., 2023). The model statistics are presented in regression tables using the *tab_model()* function of the R sjPlot package (Lüdecke, 2024).

All regression analyses were assessed for outliers using studentized residuals. Observations with studentized residuals exceeding an absolute value of 3 were considered outliers and excluded from the final regression analysis.

## Linear model for the estimation of walking speeds in the standardized parcours

We fitted a linear model (estimated using ordinary least squares (OLS)) to predict walking speed with group [NDS, SCI, stroke] (formula: speed ~ group). The model explains a statistically significant and substantial proportion of variance (R2 = 0.59, F(2, 44) = 32.14, p < .001, adj. R2 = 0.58). The model's intercept, corresponding to group = NDS, is at 1.42 (95% CI [1.33, 1.51], t(44) = 31.95, p < .001). Within this model:

- The effect of group [SCI] is statistically significant and negative (beta = -0.81, 95% CI [-1.02, -0.60], t(44) = -7.68, p < .001; Std. beta = -2.47, 95% CI [-3.12, -1.82])

- The effect of group [stroke] is statistically significant and negative (beta = -0.29, 95% CI [-0.42, -0.16], t(44) = -4.39, p < .001; Std. beta = -0.89, 95% CI [-1.29, -0.48])

Standardized parameters were obtained by fitting the model on a standardized version of the dataset. 95% Confidence Intervals (CIs) and p-values were computed using a Wald t-distribution approximation.

Model summary table:


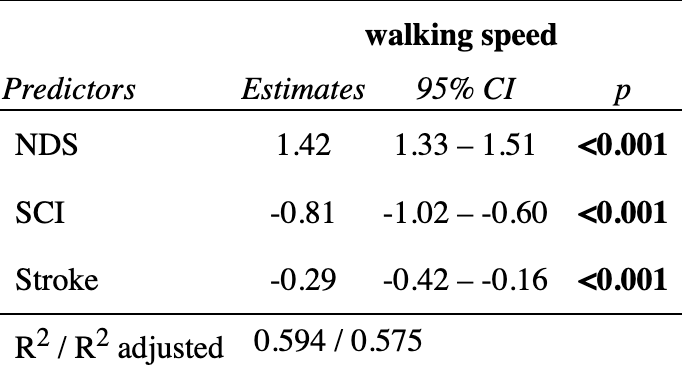


Post hoc pairwise comparisons:

| contrast | estimate | SE | df | t.ratio | p.value |
| --- | --- | --- | --- | --- | --- |
| NDS-SCI | 0.808 | 0.1050 | 44 | 7.679 | <.0001 |
| NDS-Stroke | 0.290 | 0.0661 | 44 | 4.390 | 0.0002 |
| SCI-Stroke | -0.518 | 0.1070 | 44 | -4.831 | <.0001 |

P value adjustment: tukey method for comparing a family of 3 estimates

## Linear model for the estimation of TUG in the standardized parcours cohort

We fitted a linear model (estimated using OLS) to predict TUG with group [NDS, SCI, stroke] (formula: TUG ~ group). The model explains a statistically significant and substantial proportion of variance (R2 = 0.55, F(2, 45) = 27.26, p < .001, adj. R2 = 0.53). The model's intercept, corresponding to group = NDS, is at 5.92 (95% CI [4.47, 7.36], t(45) = 8.25, p < .001). Within this model:

- The effect of group [SCI] is statistically significant and positive (beta = 10.68, 95% CI [7.20, 14.16], t(45) = 6.18, p < .001; Std. beta = 2.09, 95% CI [1.41, 2.77])

- The effect of group [Stroke] is statistically significant and positive (beta = 6.03, 95% CI [3.86, 8.20], t(45) = 5.59, p < .001; Std. beta = 1.18, 95% CI [0.75, 1.60])

Standardized parameters were obtained by fitting the model on a standardized version of the dataset. 95% Confidence Intervals (CIs) and p-values were computed using a Wald t-distribution approximation.

Model summary table:


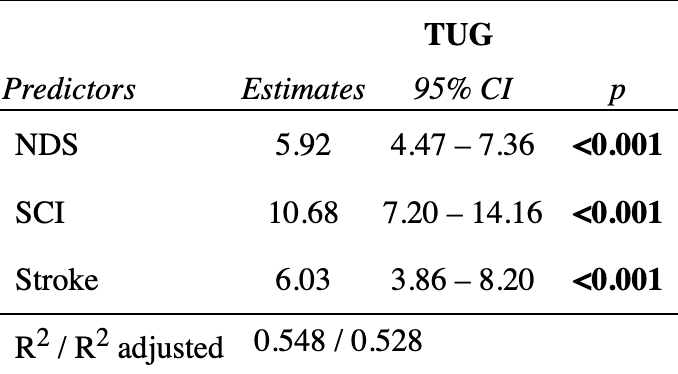


Post hoc pairwise comparisons:

| contrast | estimate | SE | df | t.ratio | p.value |
| --- | --- | --- | --- | --- | --- |
| NDS - SCI | -10.68 | 1.73 | 45 | -6.183 | <.0001 |
| NDS - Stroke | -6.03 | 1.08 | 45 | -5.587 | <.0001 |
| SCI - Stroke | 4.65 | 1.77 | 45 | 2.633 | 0.0304 |

P value adjustment: tukey method for comparing a family of 3 estimates

## Mixed linear effects model - Sensor precision in the standardized parcours cohort

### Effect of mobility type:

We fitted a linear mixed model (estimated using REML and nloptwrap optimizer) to predict the sensor-based relative deviation (RELDEV) with GROUPM [EW, MW, WALK] and parcours length (DISTANCE) (formula: RELDEV ~ 1 + GROUPM + DISTANCE). The model included the subject identifier (SUBJID) as random effect (formula: ~1 | SUBJID). The model's total explanatory power is substantial (conditional R2 = 0.75) and the part related to the fixed effects alone (marginal R2) is of 0.65. The model's intercept, corresponding to GROUPM = EW and DISTANCE = 0, is at -11.36 (95% CI [-21.52, -1.20], t(75) = -2.23, p = 0.029). Within this model:

- The effect of GROUPM [MW] is statistically non-significant and negative (beta = -3.42, 95% CI [-7.14, 0.30], t(75) = -1.83, p = 0.071; Std. beta = -0.47, 95% CI [-0.97, 0.04])

- The effect of GROUPM [WALK] is statistically significant and negative (beta = -11.75, 95% CI [-15.63, -7.87], t(75) = -6.03, p < .001; Std. beta = -1.60, 95% CI [-2.13, -1.07])

- The effect of DISTANCE is statistically significant and positive (beta = 0.06, 95% CI [0.02, 0.10], t(75) = 3.14, p = 0.002; Std. beta = 0.25, 95% CI [0.09, 0.41])

Standardized parameters were obtained by fitting the model on a standardized version of the dataset. 95% Confidence Intervals (CIs) and p-values were computed using a Wald t-distribution approximation.

Model summary table:


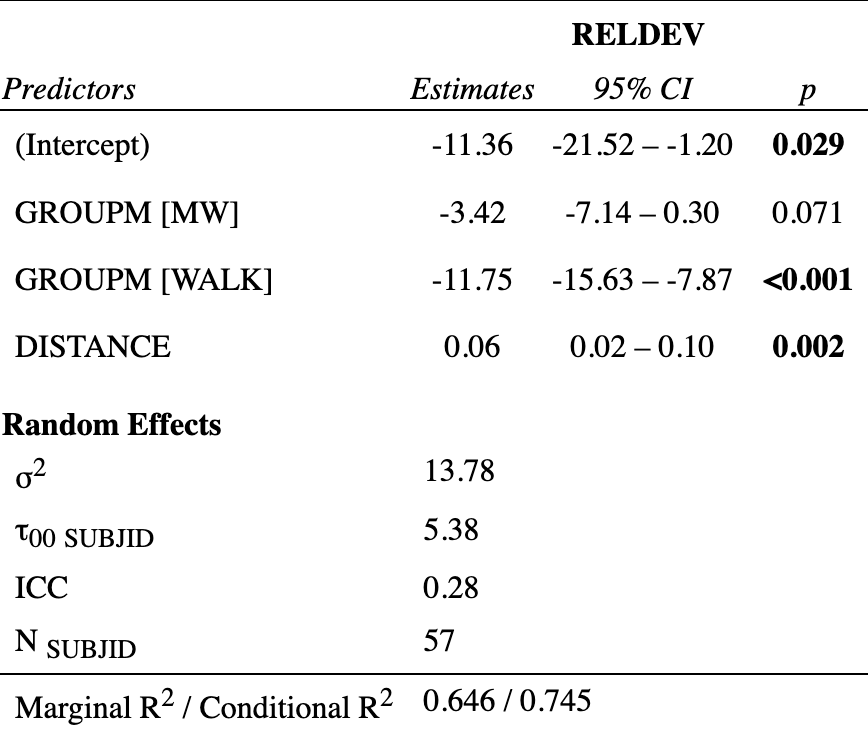


Post hoc comparisons (difference from zero)

| GROUPM | estimate | SE | df | lower.CL | upper.CL | p.value |
| --- | --- | --- | --- | --- | --- | --- |
| EW | 1.92 | 1.76 | 75.8 | -1.59 | 5.43 | 0.2795 |
| MW | -1.50 | 1.03 | 62.2 | -3.55 | 0.550 | 0.1487 |
| WALK | -9.83 | 0.695 | 68.0 | -11.2 | -8.44 | <0.0001 |

### Effect of study population and mobility type in walking individuals:

We fitted a linear mixed model (estimated using REML and nloptwrap optimizer) to predict RELDEV with GROUPM, GROUPD and DISTANCE (formula: RELDEV ~ 1 + GROUPM * GROUPD + DISTANCE). The model included SUBJID as random effect (formula: ~1 | SUBJID). The model's total explanatory power is substantial (conditional R2 = 0.80) and the part related to the fixed effects alone (marginal R2) is of 0.67. The model's intercept, corresponding to GROUPM = EW, GROUPD = HS and DISTANCE = 0, is at -13.03 (95% CI [-23.91, -2.15], t(72) = -2.39, p = 0.020). Within this model:

- The effect of GROUPM [MW] is statistically significant and negative (beta = -10.07, 95% CI [-15.70, -4.44], t(72) = -3.56, p < .001; Std. beta = -1.37, 95% CI [-2.14, -0.60])

- The effect of GROUPM [WALK] is statistically significant and negative (beta = -15.70, 95% CI [-20.58, -10.83], t(72) = -6.42, p < .001; Std. beta = -2.14, 95% CI [-2.81, -1.48])

- The effect of GROUPD [SCI] is statistically significant and negative (beta = -5.10, 95% CI [-9.51, -0.69], t(72) = -2.31, p = 0.024; Std. beta = -0.70, 95% CI [-1.30, -0.09])

- The effect of GROUPD [Stroke] is statistically non-significant and positive (beta = 0.88, 95% CI [-2.20, 3.97], t(72) = 0.57, p = 0.569; Std. beta = 0.12, 95% CI [-0.30, 0.54])

- The effect of DISTANCE is statistically significant and positive (beta = 0.09, 95% CI [0.04, 0.13], t(72) = 4.03, p < .001; Std. beta = 0.36, 95% CI [0.18, 0.55])

- The effect of GROUPM [MW] × GROUPD [SCI] is statistically significant and positive (beta = 7.94, 95% CI [2.97, 12.92], t(72) = 3.18, p = 0.002; Std. beta = 1.08, 95% CI [0.40, 1.76])

Standardized parameters were obtained by fitting the model on a standardized version of the dataset. 95% Confidence Intervals (CIs) and p-values were computed using a Wald t-distribution approximation.

Model summary table:


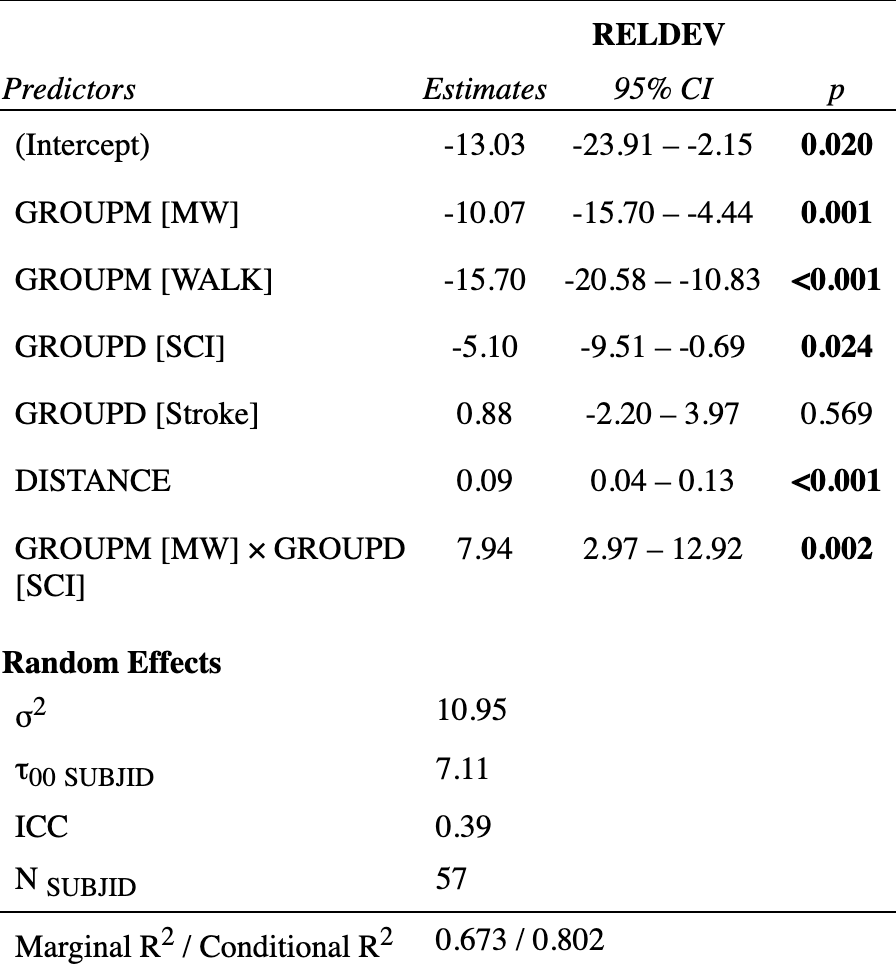


Post hoc comparisons (difference from zero; see figure 1B and table 2 of the manuscript)

| GROUPD | GROUPM | emmean | SE | df | t.ratio | p.value |
| --- | --- | --- | --- | --- | --- | --- |
| HS | EW | nonEst | NA | NA | NA | NA |
| SCI | EW | 1.08 | 1.74 | 73.7 | 0.620 | 0.5370 |
| Stroke | EW | nonEst | NA | NA | NA | NA |
| HS | MW | -3.88 | 1.43 | 71.4 | -2.709 | 0.0084 |
| SCI | MW | -1.04 | 1.32 | 71.2 | -0.792 | 0.4309 |
| Stroke | MW | nonEst | NA | NA | NA | NA |
| HS | WALK | -9.52 | 1.02 | 36.2 | -9.343 | <.0001 |
| SCI | WALK | -14.62 | 2.00 | 71.2 | -7.327 | <.0001 |
| Stroke | WALK | -8.64 | 1.17 | 69.2 | -7.407 | <.0001 |

Post hoc pairwise comparisons (in walking individuals):

| contrast | estimate | SE | df | t.ratio | p.value |
| --- | --- | --- | --- | --- | --- |
| NDS - SCI | 5.102 | 2.24 | 73.7 | 2.277 | 0.0257 |
| NDS - Stroke | -0.885 | 1.55 | 68.2 | -0.571 | 0.5696 |
| SCI - Stroke | -5.987 | 2.50 | 63.9 | -2.393 | 0.0196 |

## Change over time of the 10MWT in the NeuroMoves Cohort

We fitted a linear mixed model (estimated using REML and nloptwrap optimizer) to predict 10MWT speed (mwtspeed) with DIAGNOSIS [SCI, Stroke] and VISITNUM [1=baseline, 2=midterm, 3=final] (formula: mwtspeed ~ 1 + DIAGNOSIS + VISITNUM). The model included SUBJID as random effect (formula: ~1 | SUBJID). The model's total explanatory power is substantial (conditional R2 = 0.86) and the part related to the fixed effects alone (marginal R2) is of 0.06. The model's intercept, corresponding to DIAGNOSIS = SCI and VISITNUM = 1, is at 0.62 (95% CI [0.48, 0.76], t(246) = 8.65, p < .001). Within this model:

- The effect of DIAGNOSIS [Stroke] is statistically significant and positive (beta = 0.17, 95% CI [7.75e-05, 0.34], t(246) = 1.97, p = 0.050; Std. beta = 0.38, 95% CI [1.73e-04, 0.75])

- The effect of VISITNUM [2] is statistically significant and positive (beta = 0.09, 95% CI [0.03, 0.14], t(246) = 3.20, p = 0.002; Std. beta = 0.20, 95% CI [0.08, 0.32])

- The effect of VISITNUM [3] is statistically significant and positive (beta = 0.17, 95% CI [0.11, 0.22], t(246) = 6.18, p < .001; Std. beta = 0.37, 95% CI [0.25, 0.49])

Standardized parameters were obtained by fitting the model on a standardized version of the dataset. 95% Confidence Intervals (CIs) and p-values were computed using a Wald t-distribution approximation.

Model summary table:


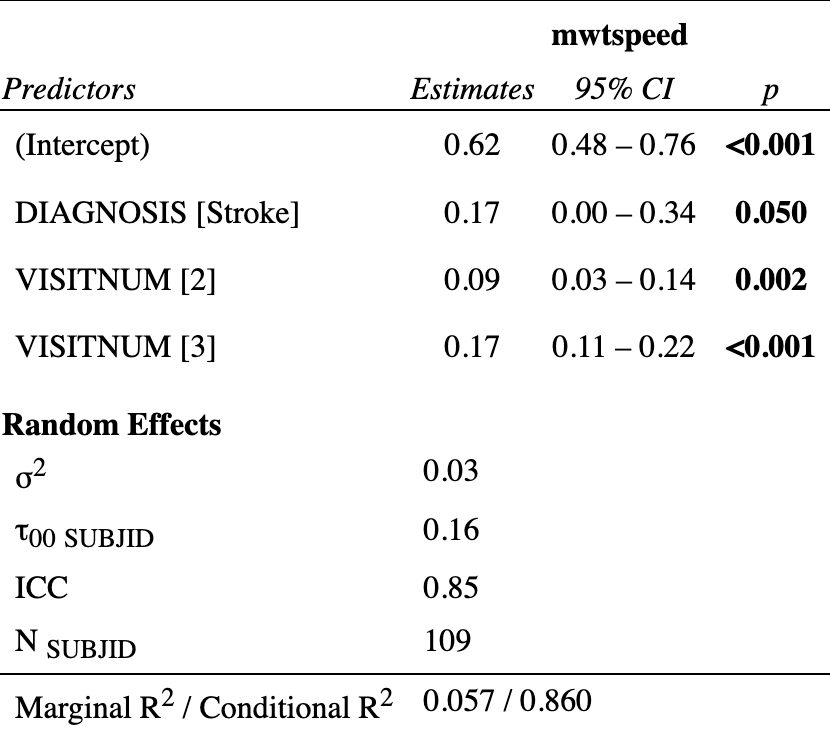


Parameter estimates according to study visit (VISITNUM (1) baseline, (2) midterm, (3) final):

| VISITNUM | emmean | SE | df | lower.CL | upper.CL |
| --- | --- | --- | --- | --- | --- |
| 1 | 0.706 | 0.0446 | 123 | 0.618 | 0.794 |
| 2 | 0.795 | 0.0469 | 146 | 0.702 | 0.888 |
| 3 | 0.873 | 0.0465 | 142 | 0.781 | 0.965 |

Post hoc pairwise comparisons:

| contrast | estimate | SE | df | t.ratio | p.value |
| --- | --- | --- | --- | --- | --- |
| midterm-baseline | 0.0890 | 0.0279 | 147 | 3.194 | 0.0017 |
| final-baseline | 0.1672 | 0.0271 | 147 | 6.172 | <.0001 |
| final-midterm | 0.0782 | 0.0288 | 143 | 2.719 | 0.0074 |

## Change over time of the TUG test in the NeuroMoves cohort

We fitted a linear mixed model (estimated using REML and nloptwrap optimizer) to predict Timed Up and Go time (tug) with DIAGNOSIS [SCI, stroke] and VISITNUM [1=baseline, 2=midterm, 3=final] (formula: tug ~ 1 + DIAGNOSIS + VISITNUM). The model included SUBJID as random effect (formula: ~1 | SUBJID). The model's total explanatory power is substantial (conditional R2 = 0.86) and the part related to the fixed effects alone (marginal R2) is of 0.06. The model's intercept, corresponding to DIAGNOSIS = SCI and VISITNUM = 1, is at 33.28 (95% CI [26.50, 40.07], t(241) = 9.66, p < .001). Within this model:

- The effect of DIAGNOSIS [Stroke] is statistically significant and negative (beta = -9.67, 95% CI [-17.76, -1.58], t(241) = -2.36, p = 0.019; Std. beta = -0.50, 95% CI [-0.91, -0.08])

- The effect of VISITNUM [2] is statistically significant and negative (beta = -4.74, 95% CI [-7.39, -2.09], t(241) = -3.53, p < .001; Std. beta = -0.24, 95% CI [-0.38, -0.11])

- The effect of VISITNUM [3] is statistically significant and negative (beta = -5.53, 95% CI [-8.08, -2.99], t(241) = -4.28, p < .001; Std. beta = -0.28, 95% CI [-0.42, -0.15])

Standardized parameters were obtained by fitting the model on a standardized version of the dataset. 95% Confidence Intervals (CIs) and p-values were computed using a Wald t-distribution approximation.

Model summary table:


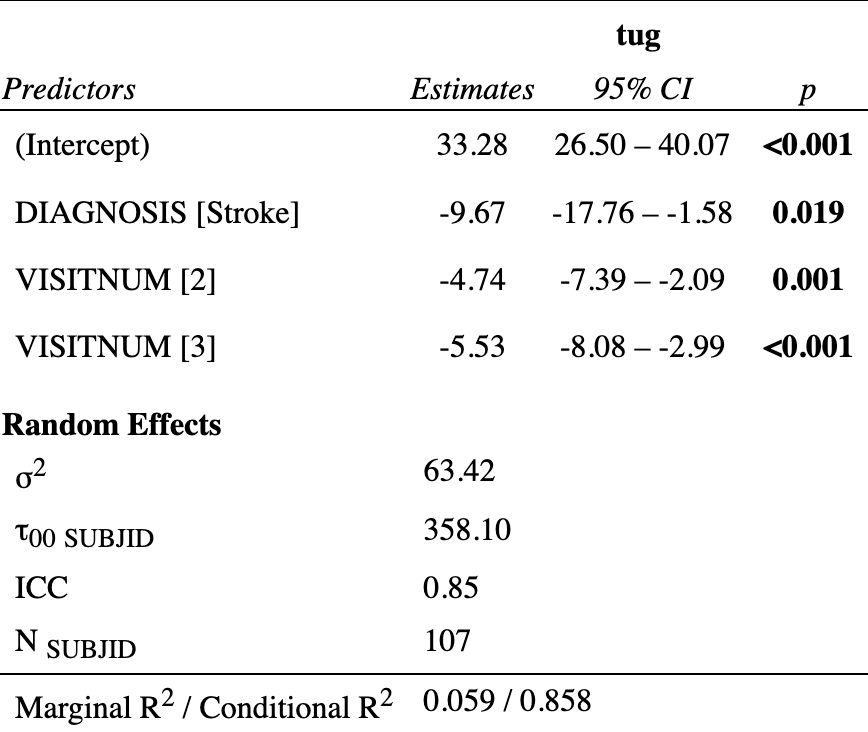


Parameter estimates according to study visit (VISITNUM (1) baseline, (2) midterm, (3) final):

| VISITNUM | emmean | SE | df | lower.CL | upper.CL |
| --- | --- | --- | --- | --- | --- |
| 1 | 28.4 | 2.14 | 122 | 24.2 | 32.7 |
| 2 | 23.7 | 2.24 | 143 | 19.3 | 28.1 |
| 3 | 22.9 | 2.22 | 139 | 18.5 | 27.3 |

Post hoc pairwise comparisons:

| contrast | estimate | SE | df | t.ratio | p.value |
| --- | --- | --- | --- | --- | --- |
| midterm-baseline | -4.740 | 1.34 | 144 | -3.526 | 0.0006 |
| final-baseline | -5.533 | 1.29 | 144 | -4.278 | <.0001 |
| final-midterm | -0.793 | 1.37 | 140 | -0.577 | 0.5651 |

## Univariate analyses of 10MWT ~ Mean Daily Distance in the NeuroMoves Cohort

As a first step, the daily walking distances for each individual were averaged over all available study days, yielding an overall average daily distance (MoverallDISTANCE) across the entire follow-up period of up to 8 months.

### Baseline Visit (VISITNUM 1)

We fitted a linear model (estimated using OLS) to predict MoverallDISTANCE with 10MWT speed (mwtspeed) (formula: MoverallDISTANCE ~ mwtspeed). The model explains a statistically significant and moderate proportion of variance (R2 = 0.26, F(1, 100) = 34.28, p < .001, adj. R2 = 0.25). The model's intercept, corresponding to mwtspeed = 0, is at 121.34 (95% CI [-130.10, 372.77], t(100) = 0.96, p = 0.341). Within this model:

- The effect of mwtspeed is statistically significant and positive (beta = 874.37, 95% CI [578.09, 1170.65], t(100) = 5.86, p < .001; Std. beta = 0.51, 95% CI [0.33, 0.68])

Standardized parameters were obtained by fitting the model on a standardized version of the dataset. 95% Confidence Intervals (CIs) and p-values were computed using a Wald t-distribution approximation.

### Midterm Visit (VISITNUM 2)

We fitted a linear model (estimated using OLS) to predict MoverallDISTANCE with mwtspeed (formula: MoverallDISTANCE ~ mwtspeed). The model explains a statistically significant and substantial proportion of variance (R2 = 0.38, F(1, 67) = 40.51, p < .001, adj. R2 = 0.37). The model's intercept, corresponding to mwtspeed = 0, is at 35.07 (95% CI [-232.94, 303.07], t(67) = 0.26, p = 0.795). Within this model:

- The effect of mwtspeed is statistically significant and positive (beta = 894.97, 95% CI [614.31, 1175.64], t(67) = 6.36, p < .001; Std. beta = 0.61, 95% CI [0.42, 0.81])

Standardized parameters were obtained by fitting the model on a standardized version of the dataset. 95% Confidence Intervals (CIs) and p-values were computed using a Wald t-distribution approximation.

### Final Visit (VISITNUM 3)

We fitted a linear model (estimated using OLS) to predict MoverallDISTANCE with mwtspeed (formula: MoverallDISTANCE ~ mwtspeed). The model explains a statistically significant and substantial proportion of variance (R2 = 0.30, F(1, 75) = 32.59, p < .001, adj. R2 = 0.29). The model's intercept, corresponding to mwtspeed = 0, is at 63.18 (95% CI [-242.56, 368.91], t(75) = 0.41, p = 0.682). Within this model:

- The effect of mwtspeed is statistically significant and positive (beta = 824.30, 95% CI [536.67, 1111.92], t(75) = 5.71, p < .001; Std. beta = 0.55, 95% CI [0.36, 0.74])

Standardized parameters were obtained by fitting the model on a standardized version of the dataset. 95% Confidence Intervals (CIs) and p-values were computed using a Wald t-distribution approximation.

## Multivariate analyses of 10MWT ~ Daily Distance in the NeuroMoves Cohort

We fitted a linear mixed model (estimated using REML and nloptwrap optimizer) to predict daily distance (DISTANCE) with 10MWT speed (mwtspeed), device unit day (DUDY) and DIAGNOSIS [SCI, Stroke] (formula: DISTANCE ~ 1 + mwtspeed * DUDY + DIAGNOSIS). The model included SUBJID as random effect (formula: ~1 | SUBJID). The model's total explanatory power is substantial (conditional R2 = 0.52) and the part related to the fixed effects alone (marginal R2) is of 0.11. The model's intercept, corresponding to mwtspeed = 0, DUDY = 0 and DIAGNOSIS = SCI, is at -159.97 (95% CI [-481.30, 161.37], t(18043) = -0.98, p = 0.329). Within this model:

- The effect of mwtspeed is statistically significant and positive (beta = 839.40, 95% CI [497.10, 1181.70], t(18043) = 4.81, p < .001; Std. beta = 0.31, 95% CI [0.19, 0.43])

- The effect of DUDY is statistically significant and positive (beta = 0.97, 95% CI [0.62, 1.32], t(18043) = 5.44, p < .001; Std. beta = 0.07, 95% CI [0.06, 0.08])

- The effect of DIAGNOSIS [Stroke] is statistically non-significant and positive (beta = 187.88, 95% CI [-107.26, 483.03], t(18043) = 1.25, p = 0.212; Std. beta = 0.17, 95% CI [-0.10, 0.44])

- The effect of mwtspeed × DUDY is statistically non-significant and positive (beta = 0.10, 95% CI [-0.31, 0.50], t(18043) = 0.46, p = 0.644; Std. beta = 2.54e-03, 95% CI [-8.26e-03, 0.01])

Standardized parameters were obtained by fitting the model on a standardized version of the dataset. 95% Confidence Intervals (CIs) and p-values were computed using a Wald t-distribution approximation.

Model summary table:


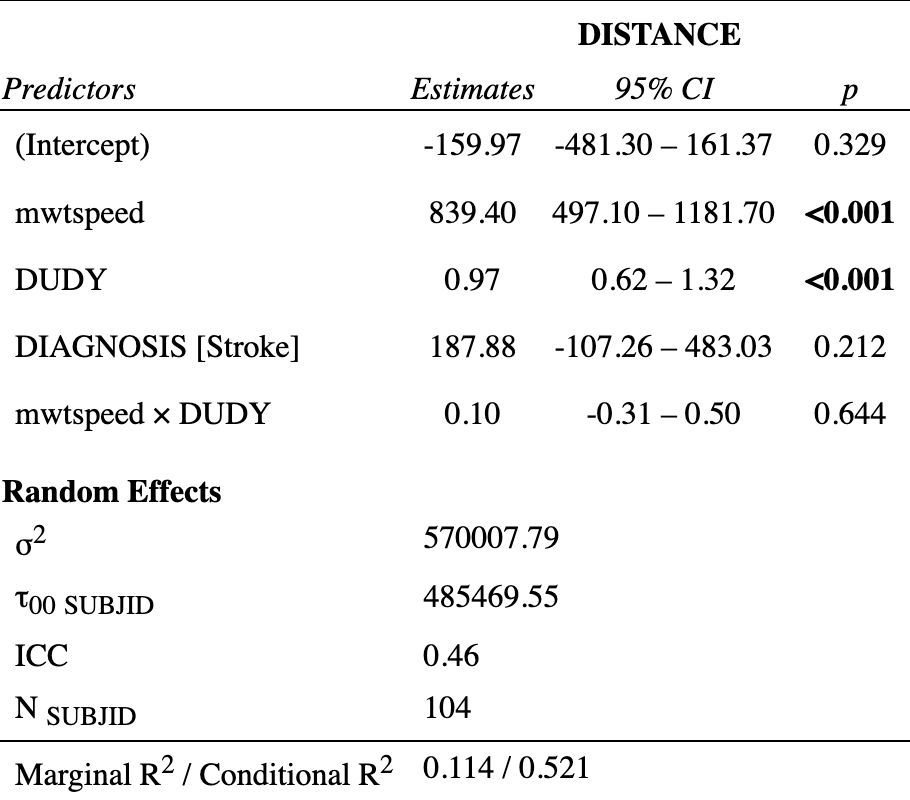


## Additional analysis of FIM ~ Daily distance in the NeuroMoves cohort

We fitted a linear mixed model (estimated using REML and nloptwrap optimizer) to predict DISTANCE with mwtspeed, functional independence measure at baseline (FIM_V1), DUDY and DIAGNOSIS [SCI, Stroke] (formula: DISTANCE ~ 1 + mwtspeed + mwtspeed:FIM_V1 + DUDY + DIAGNOSIS). The model included SUBJID as random effect (formula: ~1 | SUBJID). The model's total explanatory power is substantial (conditional R2 = 0.52) and the part related to the fixed effects alone (marginal R2) is of 0.13. The model's intercept, corresponding to mwtspeed = 0, FIM_V1 = 0, DUDY = 0 and DIAGNOSIS = SCI, is at -7.26 (95% CI [-347.59, 333.06], t(18045) = -0.04, p = 0.967). Within this model:

- The effect of mwtspeed is statistically non-significant and negative (beta = -3137.14, 95% CI [-6476.33, 202.04], t(18045) = -1.84, p = 0.066; Std. beta = 0.31, 95% CI [0.18, 0.43])

- The effect of DUDY is statistically significant and positive (beta = 1.04, 95% CI [0.88, 1.20], t(18045) = 12.68, p < .001; Std. beta = 0.07, 95% CI [0.06, 0.08])

- The effect of DIAGNOSIS [Stroke] is statistically non-significant and positive (beta = 217.58, 95% CI [-72.21, 507.37], t(18045) = 1.47, p = 0.141; Std. beta = 0.17, 95% CI [-0.10, 0.44])

- The effect of mwtspeed × FIM V1 is statistically significant and positive (beta = 32.62, 95% CI [5.44, 59.80], t(18045) = 2.35, p = 0.019; Std. beta = -0.02, 95% CI [-0.11, 0.07])

Standardized parameters were obtained by fitting the model on a standardized version of the dataset. 95% Confidence Intervals (CIs) and p-values were computed using a Wald t-distribution approximation.

Model summary table:


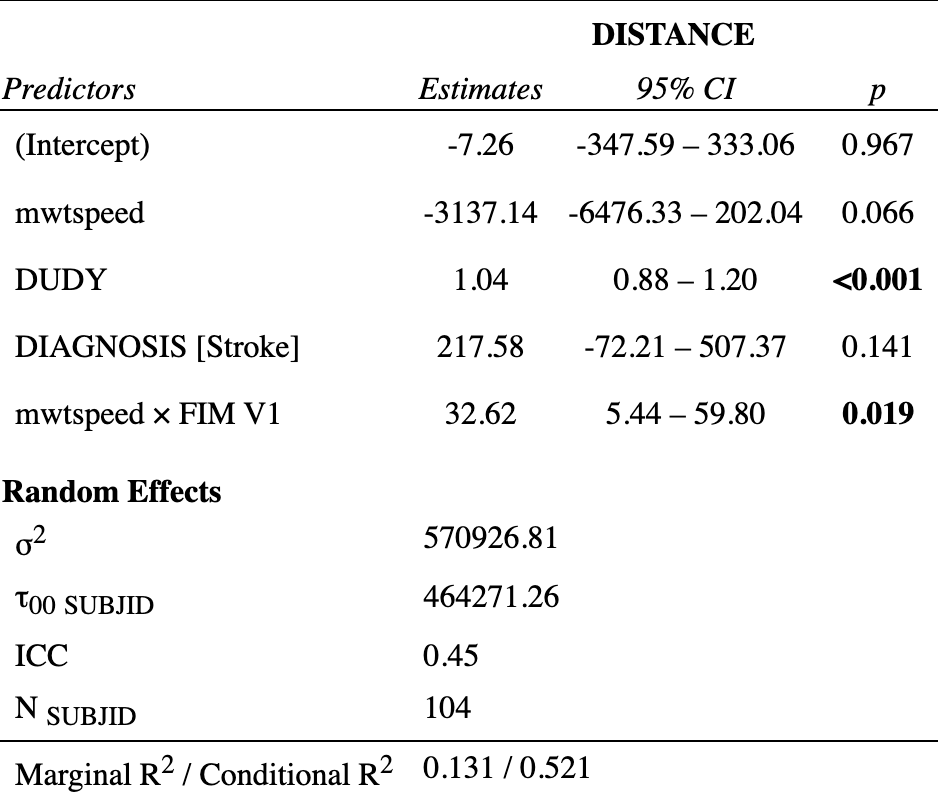


# References

Lüdecke, D. (2024). *sjPlot: Data visualization for statistics in social science* [Manual]. https://CRAN.R-project.org/package=sjPlot

Makowski, D., Lüdecke, D., Patil, I., Thériault, R., Ben-Shachar, M. S., & Wiernik, B. M. (2023). Automated results reporting as a practical tool to improve reproducibility and methodological best practices adoption. *CRAN*. https://easystats.github.io/report/
